# The Impact and Reliability of Tissue Segmentation on In Vivo Magnetic Resonance Spectroscopy Metabolite Quantification

**DOI:** 10.1002/mrm.70380

**Published:** 2026-04-10

**Authors:** Jessica Archibald, Kay Chioma Igwe, Antonia Kaiser, Karl Landheer, Jaimie Lee, John L. K. Kramer, Aaron T. Gudmundson, Helge J. Zöllner, Georg Oeltzschner, Candace C. Fleischer, Niklaus Zölch, Jamie Near, Mark Mikkelsen

**Affiliations:** ^1^ Department of Radiology Weill Cornell Medicine New York New York USA; ^2^ Department of Biomedical Engineering Columbia University Fu Foundation School of Engineering and Applied Science New York New York USA; ^3^ CIBM Center for Biomedical Imaging École Polytechnique Fédérale de Lausanne Lausanne Switzerland; ^4^ Regeneron Genetics Center Tarrytown New York USA; ^5^ Department of Anesthesiology, Pharmacology and Therapeutics, Faculty of Medicine University of British Columbia Vancouver British Columbia Canada; ^6^ The Malone Center for Engineering in Healthcare Johns Hopkins University Baltimore Maryland USA; ^7^ Russell H. Morgan Department of Radiology and Radiological Science Johns Hopkins University School of Medicine Baltimore Maryland USA; ^8^ Department of Radiology and Imaging Sciences Emory University School of Medicine Atlanta Georgia USA; ^9^ Department of Biomedical Engineering Georgia Institute of Technology and Emory University Atlanta Georgia USA; ^10^ Institute of Forensic Medicine Universität Zürich Zürich Switzerland; ^11^ Sunnybrook Research Institute and University of Toronto Toronto Ontario Canada

**Keywords:** ANTs, FSL, magnetic resonance spectroscopy, metabolite quantification, SPM, tissue segmentation

## Abstract

**Purpose:**

Quantification of metabolite concentrations using MRS requires tissue‐dependent signal corrections. Accurate estimation of voxel tissue composition is therefore essential. Commonly used brain tissue segmentation tools differ in their algorithms and implementation, potentially introducing variability in MRS‐derived concentration estimates. This study investigates the impact and reliability of tissue segmentation on metabolite quantification.

**Methods:**

Three segmentation tools (ANTs, FSL, SPM) were evaluated using an in vivo test–retest MRI/MRS dataset. Voxelwise GM/WM/CSF fractions were applied to compute tissue‐corrected total creatine (tCr) concentrations. Linear mixed‐effects modeling, variance‐component partitioning, and intraclass correlation coefficients (ICCs) quantified tool‐, session‐, and participant‐related variance under permutation scenarios that isolated segmentation‐ and MRS‐related effects. As a benchmark for segmentation performance, comparisons with manually segmented data were conducted across three brain regions.

**Results:**

Segmentation tools produced systematically different tissue fractions that propagated into differences in tCr concentration estimates. Variance partitioning attributed 56.8%, 50.0%, and 51.3% of total tCr concentration variability to segmentation tool across the three permutations, with participant‐specific factors accounting for 34.7%, 36.2%, and 28.5%, respectively. When segmentation variability was held constant, test–retest reliability was high (ICC > 0.8) but dropped to ∼0.5 when both segmentation and MRS variability varied. Agreement with manual segmentation was region‐ and tool‐dependent, with the lowest agreement in the thalamus.

**Conclusion:**

Tissue segmentation contributes substantially to the variability in MRS‐derived metabolite concentration estimates. These results underscore the need for transparent segmentation reporting and data sharing to ensure reproducibility and cross‐study comparability in MRS research.

## 1 Introduction

MRS is a non‐invasive spectroscopic technique used to assess the chemical composition of a biological sample [1]. By estimating metabolite concentrations in vivo, MRS provides insights into normal and pathological biological processes throughout the body. Its application to the human brain has yielded major insights into sensory processing mechanisms [2, 3, 4, 5], brain development [6], chronic pain conditions [7, 8], neurodegenerative diseases [9, 10], psychiatric disorders [11], and metabolic changes associated with aging [12]. For MRS to support clinical decision‐making, however, it is critical to minimize methodological variability that can obscure or inflate intrinsic physiological effects. “Absolute” concentration estimates in biochemical units are increasingly favored due to their biological interpretability and potential for cross‐study comparisons [13].

Expert consensus has identified methodological variability spanning data acquisition, preprocessing, and quantification as a key barrier to the clinical translation of MRS [14]. Several factors contribute to quantification variability, including differences in relaxation correction [15] and modeling approaches [16]. Tissue segmentation is another major source of variance, as it is required for the tissue correction used in water‐referenced MRS quantification. Although water‐referenced MRS quantification relies on several assumptions beyond the scope of the present study, such as water content and relaxation properties [13, 15, 16, 17], these calculations depend heavily on accurate estimates of gray matter (GM), white matter (WM), and cerebrospinal fluid (CSF) fractions within MRS volumes of interest. Previous work at 1.5 T has demonstrated that variability in image segmentation approaches, particularly in partial‐volume estimates of CSF and GM, can substantially impact metabolite concentration estimates [15]. For example, differences of up to 14% in NAA have been reported depending on the segmentation tool used with multispectral imaging data [15].

Accurate tissue segmentation derived from high‐resolution anatomical MRI scans is therefore essential for reliably estimating metabolite concentrations. While GM and WM fractions inform the application of appropriate signal relaxation corrections, the CSF fraction has a particularly strong influence on final concentration estimates due to its assumed negligible metabolite signal [13] and vastly different relaxation properties compared to GM and WM [18, 19]. Numerous automated software packages are publicly available for brain tissue segmentation, specifically for GM, WM, and CSF; however, each package applies different mathematical and analytical approaches for tissue classification and, in some cases, their use of prior knowledge (e.g., from a brain template or atlas), introducing a wide range of segmentation differences [20, 21, 22]. For example, Advanced Normalization Tools (ANTs) combines Markov random field (MRF) theory with template‐based priors to inform tissue classification [21]. In contrast, the FMRIB Software Library (FSL) relies on hidden Markov random field (HMRF) models, where tissue class membership is determined by both voxel intensity and the contextual constraint of neighboring voxels [22]. Finally, Statistical Parametric Mapping (SPM) uses a combination of a brain prior and a series of Gaussian mixture models (GMMs) to perform tissue segmentation [20].

Comparative evaluations have shown that automated segmentation algorithms yield systematically different tissue classifications [15, 23], including when tested against the simulated BrainWeb MRI dataset [24] and manual segmentation [25]. Kazemi and Noorizadeh [26], reported that SPM8 achieved the highest accuracy relative to ground truth (e.g., simulated data) when compared against FSL and Brainsuite. Further, Katuwal et al. showed SPM had the closest estimates to manual segmentation [25]. Yet discrepancies between packages reach the same order of magnitude as volumetric changes typically observed in neurological diseases [24, 27], highlighting that segmentation uncertainty may give rise to apparent effects that resemble true biological changes.

This study examined how tissue segmentation and its associated errors impact MRS metabolite quantification by assessing the impact and reliability of several segmentation tools. In one experiment, we used an in vivo test–retest MRI/MRS dataset. By applying outputs from three commonly used segmentation software tools (i.e., ANTs, FSL, and SPM) to corresponding MRS data, we quantified how differences in tissue fraction estimates propagate into estimated absolute metabolite concentrations. We also investigated how variance was apportioned to the effects of segmentation tool, MRS measurement, and individual differences. In a second experiment, we used the MRBrainS dataset [28], a 3 T structural MRI benchmark with expertly manually labeled GM/WM/CSF tissue classes, to evaluate automated tissue segmentation across multiple MRS volumes of interest.

## 2 Methods

### 2.1 Experiment 1: In Vivo Test–Retest Dataset

**FIGURE 1:**
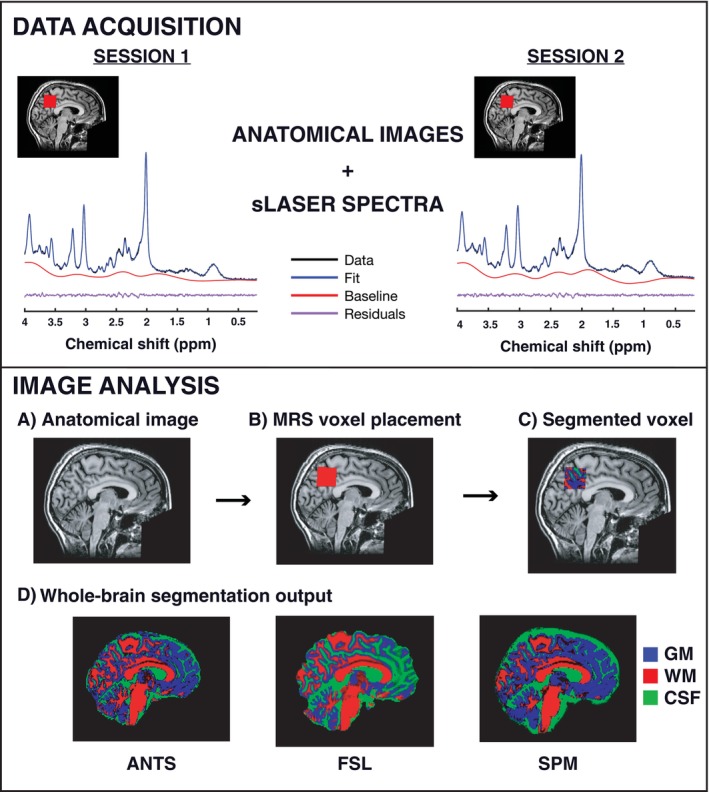
Experiment 1 study design of in vivo MR data acquisition and analysis. A 27‐mL voxel was positioned in the medial parietal lobe to localize MRS sLASER data. A representative spectrum from one participant across two scan sessions, including the raw spectral data (black), LCModel fit (blue), baseline estimate (red), and residuals (purple). Brain extraction and tissue segmentation were performed using three software tools: ANTs, FSL, and SPM.

Sixteen healthy adults (6 males, 10 females; mean age ± SD: 38.4 ± 18.2 range: 19–66 years) were enrolled in a separate study focused on test–retest reliability [29]. Ethical approval for this study was obtained from the Weill Cornell Medicine Institutional Review Board (protocol #0807009883), and all participants provided written informed consent before their participation. Each participant completed two MR scan sessions (henceforth referred to as session 1 and session 2), with a median interval of 0 days between sessions (range: 0–29 days), as most were scanned on the same day; only one participant had the longest interval (29 days). The exclusion criteria were for individuals with contraindications to MRI or a history of neurological or psychiatric disorders. The study design, including MRI/MRS data acquisition and segmentation workflow, is illustrated in Figure 1.

#### 2.1.1 MR Scanning Protocol

Data were collected on a 3 T GE Discovery MR750 scanner using a ^1^H 32‐channel phased‐array RF head coil for receive and a body coil for transmit. High‐resolution 3D *T*_1−_weighted BRAVO structural scans (FSPGR; TR/TE/TI = 12.2/5.2/725 ms; flip angle = 7°; voxel resolution = 0.9 × 0.9 × 1.5 mm^3^; matrix size = 256 × 256; slices = 124; parallel acceleration factor = 2) were first acquired for accurate voxel placement in each scan session. Single‐voxel MRS data were acquired using sLASER [30] with TE/TR = 35/2000 ms, a spectral width of 5000 Hz, 4096 data points, and 64 transients. The MRS voxel (resolution = 3 × 3 × 3 cm^3^) was placed in the medial parietal lobe (Figure 1). Water suppression was performed using VAPOR [31]. All details for the MRS acquisition and analysis can be found in the MRSinMRS table (Table S2) [14].

#### 2.1.2 MR Image Segmentation

The 3D *T*_1_‐weighted structural data were brain‐extracted and segmented into GM, WM, and CSF maps using ANTs, FSL, and SPM. Brain extraction was performed separately within each segmentation software to maintain consistency with its native segmentation workflow. For comparability across tools, tissue‐fraction maps were verified to sum to 1 within each MRS voxel and were normalized when necessary. Additional methodological details are provided in the Supporting Information S1.

#### 2.1.3 MRS Data Processing

MRS data were processed in Osprey [32] (v2.9.5) and fitted using the embedded LCModel [33] wrapper (v6.3‐1N). A sLASER basis set of 20 metabolites was generated using FID‐A [34] with the following simulation parameters: alanine (Ala), ascorbate (Asc), aspartate (Asp), creatine (Cr), γ‐aminobutyric acid (GABA), glucose (Glc), glutamine (Gln), glutamate (Glu), glycine (Gly), glycerophosphocholine (GPC), glutathione (GSH), *myo*‐inositol (mI), lactate (Lac), *N*‐acetylaspartate (NAA), *N*‐acetylaspartylglutamate (NAAG), phosphocholine (PCh), phosphocreatine (PCr), phosphoethanolamine (PE), *scyllo*‐inositol (scyllo), and taurine (tau), a linewidth of 2 Hz, a spectral width of 5000 Hz, and 64 × 64 spatial grid points [35]. The metabolite and water signals were then used to calculate the molar concentrations of total NAA (tNAA), total Cr (tCr), total Cho (tCho), mI, Glu, and the combination of Glu and Gln (Glx) [16]. We report results for tCr in the main text. Results for the remaining metabolites are presented in the Supporting Information S1. This choice was made because segmentation‐related variability propagates equivalently across all metabolites.

#### 2.1.4 Metabolite Quantification

Anatomical segmentation variability was evaluated using three software packages (ANTs, FSL, and SPM) by calculating fractional volumes of GM, WM, and CSF within the MRS voxel. The varying tissue fractions were used to compute millimolar (mM) metabolite concentration [*M*]_molar_ according to Gasparovic et al. [13, 36]

$$\begin{array}{ll} & {\left[M\right]}_{\text{molar}}\\ & \;=\frac{S_{M,\mathrm{obs}}\left(f_{\mathrm{GM}}d_{\mathrm{GM}}R_{H2O,\mathrm{GM}}+f_{\mathrm{WM}}d_{\mathrm{WM}}R_{H2O,\mathrm{WM}}+f_{\mathrm{CSF}}d_{\mathrm{CSF}}R_{H2O,\mathrm{CSF}}\right)}{S_{H2O,\mathrm{obs}}\;\left(1-f_{\mathrm{CSF}}\right)R_{M}}{\left[H_{2}O\right]}_{\text{molar}} \end{array}$$

where

- *S*_M,obs_ and *S*_H2O,obs_ are the observed metabolite and water signals;
- *f*_*x*_ is the fractional volume of GM, WM, and CSF within the voxel;
- *d*_*x*_ is the density of water in GM, WM, and CSF (*d*_GM_ = 0.78, *d*_WM_ = 0.65, and *d*_CSF_ = 0.97) [13, 17];
- *R*_H2O,*x*_ is the relaxation attenuation factor for water in GM, WM, and CSF;
- *R*_M_ is a scaling factor that accounts for the relaxation times of metabolite protons averaged across GM and WM [36, 37].
- [H_2_O]_molar_ is the millimolar concentration of pure water (55.51 × 10^3^ mmol/L [13]).

Relaxation attenuation terms were calculated using a standard exponential model [13, 36], $R=\exp\left(-\mathrm{TE}/T_{2}\right)\left[1-\exp\left(-\mathrm{TR}/T_{1}\right)\right]$, where water‐specific *T*_1_ and *T*_2_ values were taken from the literature for GM, WM, and CSF [38]. For metabolites, we used *T*_1_ and *T*_2_ values from prior studies averaged across GM and WM [37, 39] (Table S3). Note that the number of protons for each metabolite is encoded in the basis set (see Data Availability Statement).

### 2.2 Experiment 1: Statistical Analysis

Statistical analyses were performed in R (v4.4.0). A significance level of *p* < 0.05 was used for all inference tests. To test if metabolite concentration values were normally distributed, Shapiro–Wilk tests were performed for each metabolite (tNAA, tCr, mI, Glu, and Glx) within each segmentation dataset for each scan session, with Bonferroni correction applied to account for multiple comparisons.

#### 2.2.1 Between‐Session Differences

Structural MRI quality metrics were derived using MRIQC [40], which computes quantitative quality indices from the *T*_1_‐weighted anatomical images to evaluate intensity uniformity, contrast, sharpness, and signal quality. The coefficient of joint variation (CJV) quantifies the ratio of within‐tissue intensity variability in GM and WM relative to the difference in their mean intensities. It serves as a proxy for intensity nonuniformity, with lower values indicating better homogeneity. The contrast‐to‐noise ratio (CNR) measures the separability of GM and WM intensity distributions, accounting for image noise, with higher values indicating greater tissue contrast. The entropy‐focus criterion (EFC) estimates the Shannon entropy of voxel intensities, normalized to the image energy, and reflects the degree of image ghosting and blurring. Lower EFC values correspond to sharper, less motion‐contaminated images. Finally, the total SNR represents the mean intensity of brain voxels relative to their variability and provides an index of overall signal quality, with higher values indicating better image quality.

It should be noted that MRIQC performs its computations using segmentation outputs derived from FSL, meaning that metrics dependent on tissue segmentation masks (CJV, CNR, and total SNR) are software‐dependent and reflect FSL's segmentation performance. In contrast, EFC is independent of segmentation masks. All quality measures were interpreted in this context, acknowledging their dependence on the underlying segmentation framework.

A paired *t*‐test was used to assess differences between the two scan sessions after confirming data normality. Quality metrics, including the tCr SNR, water peak full‐width at half‐maximum (H_2_O FWHM), and the fit quality index (defined as the ratio of the sum of squares of residuals to the variance of the noise), were evaluated for session‐related differences.

#### 2.2.2 Linear Mixed‐Effects Modeling

To evaluate the effects of segmentation software tool on composition estimates of GM, WM, and CSF, repeated‐measures linear mixed‐effects models were fitted separately using the *nlme* package [41], as follows: lme(TissueFraction ∼ SoftwareTool * Session, random = ∼1 | Participant, data). Each model included software tool, session, and their interaction as fixed effects, with participants modeled as a random effect to account for repeated measures. Post hoc pairwise comparisons were performed using Tukey's HSD.

To assess whether segmentation software tool and session influence metabolite quantification, repeated‐measures linear mixed‐effects models were similarly fitted separately for each metabolite (tNAA, tCr, Glu, Glx, and mI are reported in the Supporting Information S1), with segmentation tool, session, and their interaction as fixed effects, and participant as a random effect. The model was specified as: lme(MetaboliteConcentration ∼ SoftwareTool * Session, random = ∼1 | Participant, data). ANOVA‐style tables summarizing the fixed effects were generated for each model. Post hoc pairwise comparisons were performed using Tukey's HSD.

#### 2.2.3 Test–Retest Reliability and Variance Partitioning

To evaluate the reliability of the different segmentation tools on estimated tCr levels and the relative contributions of segmentation and spectral fitting/quantification variability, three test–retest permutations were implemented. For each permutation, within‐ and between‐subjects coefficients of variation (CV_ws_ and CV_bs_) and single‐measure intraclass correlation coefficients (ICCs; two‐way mixed model, absolute agreement) [42] were computed to quantify reliability. CVs and ICCs were compared across segmentation tools to assess their relative sensitivity to segmentation‐ and fitting/quantification‐related effects. To determine whether any tool was significantly more reliable than another, we computed one‐tailed *p*‐values for the previously calculated ICCs for each tool, with the null hypothesis (*H*_0_) set to the ICC of a compared tool. For example, to determine whether SPM was more reliable than ANTs, we computed the *p*‐value for the ICC for SPM by setting *H*_0_ to the ICC for ANTs.

Variance partition coefficients (VPC) were estimated using linear mixed‐effects models to quantify the proportion of total variability attributable to each random effect in the analysis pipeline, following Mikkelsen et al. [43]. Specifically, the models partitioned variance into components associated with the segmentation tool, session, participant, and residual error. This allowed us to characterize the extent to which the total variance was driven by biological between‐subject differences versus methodological or session‐dependent differences.

Three test–retest permutations were used to isolate distinct sources of within‐subject variance:

1. *Structural‐only permutation*: MRS data from session 1 were quantified using segmentation maps from both sessions, isolating the effect of segmentation variability.
2. *MRS‐only permutation*: both MRS sessions were quantified using the segmentation output from session 1, isolating variability arising from spectral fitting and quantification as well as session‐to‐session physiological state differences.
3. *Combined permutation*: session‐specific MRS and MRI data were paired to capture the total test–retest variability across the full analysis pipeline (i.e., a “conventional” analysis).

Bland–Altman analysis was used to visualize both test–retest reliability and between‐tool agreement. For test–retest comparisons, differences in tissue‐corrected tCr estimates between sessions 1 and 2 were plotted against their mean for each segmentation tool. For between‐tool comparisons, pairwise differences in tissue‐corrected tCr concentrations were plotted.

Voxelwise spatial overlap between MRS voxels from sessions 1 and 2 was quantified using the Dice overlap coefficient [44], defined as twice the intersection volume of the two voxel masks divided by the sum of their individual volumes. This metric provides a symmetric measure of spatial agreement, ranging from 0 (no overlap) to 1 (perfect alignment), and was used as an index of voxel placement reproducibility. Voxel masks were extracted from MRS co‐registration outputs in Osprey and resampled to MNI152 space using SPM12.

### 2.3 Experiment 2: MRBrainS Dataset

3D *T*_1_‐weighted structural data were sourced from a public 3 T Philips Achieva MR dataset (TE/TR = 4.5/7.9 ms) from University Medical Center Utrecht [28], involving 20 subjects (10 males, 10 females; aged 65–80 years, mean ± SD = 71 ± 4 years) from a cohort of functionally independent individuals with no history of brain disease. These data included manual segmentation. In addition, tissue segmentation was performed using ANTs, FSL, and SPM, as in Experiment 1 (see Section 2.1.2), and was compared with manual segmentation, which served as the reference against which the other approaches were compared.

#### 2.3.1 Synthetic MRS ROIs

Binary voxels were made in ITK‐SNAP toolbox (V.4.0.2) in three brain regions: the anterior cingulate cortex (ACC), occipital cortex (OCC), and left thalamus (LThalamus). To ensure identical ROI dimensions in all downstream analyses, we regenerated standardized 20‐mm isotropic ROIs centered on each voxel. Relative tissue volume fractions from each segmentation approach were then extracted within these ROIs. In addition, a normalized GM fraction was computed, $t_{\mathrm{GM}}=\frac{f_{\mathrm{GM}}}{f_{\mathrm{GM}}+f_{\mathrm{WM}}}$, to summarize GM/WM composition per unit tissue volume to facilitate interpretation.

### 2.4 Experiment 2: Statistical Analysis

To assess robustness to manual‐label bias, an ancillary analysis included a “perturbed” version of each manual mask, in which 5% of voxels within the ROIs were randomly reassigned to a different tissue class.

Manual segmentation was treated as the reference for each region/tissue combination. For each segmentation tool, single‐measure, two‐way mixed‐model ICCs (absolute agreement) were computed between the tool's tissue‐fraction estimates and the manual segmentation. ICCs from the original and perturbed manual masks were compared using Zou's confidence‐interval method [45]. A Bonferroni‐adjusted threshold was used to control for multiple comparisons.

### 2.5 Exploratory Analysis: Effect of Defacing Structural Images

Defacing is routinely mandated when sharing structural MR images to safeguard participant privacy, yet recent work has shown that the operation impacts quantitative assessments of brain images. Bhalerao et al. reported that four widely used defacing approaches significantly altered volumetric and image quality measures [46], whereas other examinations reported a weaker effect [47].

Since defacing is necessary for openly sharing our data, we performed an exploratory comparison of tissue‐corrected metabolite concentrations obtained from native and defaced anatomical images using the ANTs, FSL, and SPM pipelines. This analysis allows us to identify segmentation tools that are robust to defacing effects and to offer evidence‐based guidance to researchers analyzing MRS datasets with defaced anatomical images. This exploratory analysis was performed on the dataset from Experiment 1, as the field of view of the data from Experiment 2 was tilted to not capture participants' faces.

## 3 Results

Data from two participants were excluded due to a lack of relevant consent for data sharing, and data from one participant were excluded due to the participant's wish to end the scan. For the remaining cohort (*n* = 13), between‐session differences were evaluated. MRS quality metrics for both sessions are summarized in Table 1. Representative axial T1‐weighted images from both datasets are provided in Figure S1. Shapiro–Wilk tests indicated no significant departures from normality in either session (all *p* > 0.05). Full results are provided in the Supporting Information S1, including density plots (Figure S2 and Table S1).

**TABLE 1:** MRS spectral quality metrics (mean ± SD).

Session	tCr SNR	H_2_O FWHM	Fit quality
1	152.43 ± 14.14	7.27 ± 0.66	2.36 ± 0.29
2	156.03 ± 13.49	7.23 ± 0.63	2.67 ± 0.59

### 3.1 Experiment 1: Data Quality Metrics

No significant session‐to‐session differences in spectral quality metrics (tCr SNR: *t* = −0.98, *p* = 0.99; H_2_O FWHM: *t* = 0.26, *p* < 0.99; fit quality index: *t* = −2.07, *p* = 0.18) were found. In addition to the MRIQC metrics, all *T*_1_‐weighted anatomical images were visually inspected by an experienced rater to confirm overall data quality. No significant session‐to‐session differences in MRIQC‐derived quality metrics were observed (CNR: session 1 = 1.44 ± 0.16, session 2 = 1.44 ± 0.15; *t* = 0.05, *p* = 0.96; total SNR: session 1 = 4.26 ± 0.39, session 2 = 4.24 ± 0.33; *t* = 0.18, *p* = 0.86; Figure S3).

### 3.2 Effect of Segmentation Tool on Tissue Fraction Estimates

**FIGURE 2:**
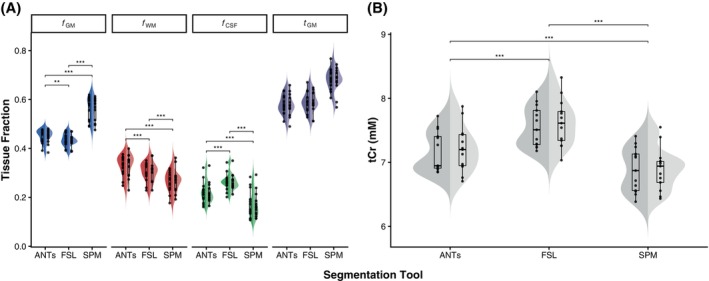
(A) Comparison of tissue segmentation output across the three software tools. Fractional gray matter (*f*_GM_), white matter (*f*_WM_), cerebrospinal fluid (*f*_CSF_), and normalized fractional GM ($t_{\mathrm{GM}}=\frac{f_{\mathrm{GM}}}{f_{\mathrm{GM}}+f_{\mathrm{WM}}}$) are shown on the *x*‐axis. Data were pooled over both sessions (*n* = 26). (B) Estimated absolute tCr concentrations (mM) tissue corrected using tissue fractions from the three tools. Statistical results are shown from repeated‐measures ANOVA with post hoc Tukey HSD‐corrected pairwise comparisons. **p < 0.01; ***p < 0.001.

For GM tissue fraction estimates, there was a significant main effect of segmentation tool (*F*(2,60) = 434.23, *p* < 0.0001), with no significant effect of session (*F* (1, 60) = 0.54, *p* = 0.47) and no significant interaction (*F* (2, 60) = 0.05, *p* = 0.95). Estimated marginal means averaged over sessions showed that SPM produced the highest GM fraction (mean = 0.56, 95% CI = [0.54, 0.58]), followed by ANTs (mean = 0.45, CI = [0.43, 0.47]) and FSL (mean = 0.43, CI = [0.41, 0.45]). Post hoc Tukey HSD‐adjusted pairwise comparisons for segmentation tool revealed that ANTs estimates were significantly higher than FSL (mean difference = 0.02, SE = 0.01, *p* = 0.002), and SPM was significantly higher than both ANTs (mean difference = 0.11, SE = 0.01, *p* < 0.001) and FSL (mean difference = 0.13, SE = 0.01, *p* < 0.001). Session means were similar (session 1: mean = 0.48, CI = [0.46, 0.50]; session 2: mean = 0.48, CI = [0.46, 0.50]), with no significant difference between sessions (mean difference = 0.00, SE = 0.01, *p* = 0.47) (Figure 2).

For WM tissue fraction estimates, there was a significant main effect of segmentation tool (*F* (2, 60) = 120.54, *p* < 0.001), with no significant effect of session (*F* (1, 60) = 0.98, *p* = 0.33) and no significant interaction (*F* (2, 60) = 0.24, *p* = 0.79). Estimated marginal means averaged over sessions showed that ANTs produced the highest WM fraction (mean = 0.33, CI [0.31, 0.36]), followed by FSL (mean = 0.30, CI = [0.28, 0.33]) and SPM (mean = 0.27, CI = [0.24, 0.29]). Post hoc pairwise comparisons for segmentation tool revealed that ANTs estimates were significantly higher than both FSL (mean difference = 0.03, SE = 0.00, *p* < 0.001) and SPM (mean difference = 0.06, SE = 0.00, *p* < 0.001), and FSL estimates were significantly higher than SPM (mean difference = 0.04, SE = 0.00, *p* < 0.001). Session means were similar (session 1: mean = 0.30, CI = [0.28, 0.32]; session 2: mean = 0.30, CI = [0.28, 0.33]), with no significant difference between sessions (mean difference = 0.00, SE = 0.00, *p* = 0.33) (Figure 2).

For CSF tissue fraction estimates, there was a significant main effect of segmentation tool (*F* (2, 60) = 185.62, *p* < 0.001), with no significant effect of session (*F* (1, 60) = 0.01, *p* = 0.90) and no significant interaction (*F* (2, 60) = 0.08, *p* = 0.93). Estimated marginal means averaged over sessions showed that FSL produced the highest CSF fraction (mean = 0.26, CI = [0.24, 0.29]), followed by ANTs (mean = 0.22, CI = [0.19, 0.25]) and SPM (mean = 0.17, CI = [0.14, 0.20]). Post hoc pairwise comparisons for segmentation tool revealed that ANTs estimates were significantly lower than FSL (mean difference = −0.04, SE = 0.00, *p* < 0.001), while ANTs estimates were significantly higher than SPM (mean difference = 0.05, SE = 0.00, *p* < 0.001), and FSL estimates were significantly higher than SPM (mean difference = 0.09, SE = 0.00, *p* < 0.001). Session means were nearly identical (session 1: mean = 0.22, CI = [0.19, 0.24]; session 2: mean = 0.22, CI = [0.19, 0.25]), with no significant difference between sessions (mean difference = 0.00, SE = 0.01, *p* = 0.90) (Figure 2).

### 3.3 Effect of Segmentation Tool on Quantified Metabolites

For quantified tCr concentrations, the analysis showed a significant main effect of segmentation tool, *F* (2, 60) = 64.63, *p* < 0.001, with no significant session effect, *F* (1, 60) = 0.59, *p* = 0.44, and no interaction (of session and segmentation tool), *F* (2, 60) = 0.06, *p* = 0.94 (ANTS: mean = 7.19, CI = [7.01, 7.37]; FSL: mean = 7.59, CI = [7.40, 7.77]; SPM: mean = 6.89, CI = [6.71, 7.07]). All post hoc pairwise comparisons between segmentation tools are provided in Table 2. Results for additional metabolites are summarized in Figure S4 and Table S4.

**TABLE 2:** Tukey HSD‐adjusted pairwise comparisons of quantified absolute tCr concentration estimates (mM) between segmentation tools.

Tool	Difference	SE	df	*t*	*p*
ANTs vs. FSL	−0.40	0.06	60	−6.48	< 0.001
ANTs vs. SPM	0.30	0.06	60	4.85	< 0.001
FSL vs. SPM	0.70	0.06	60	11.33	< 0.001

### 3.4 Test–Retest Reliability and Variance Partitioning

**FIGURE 3:**
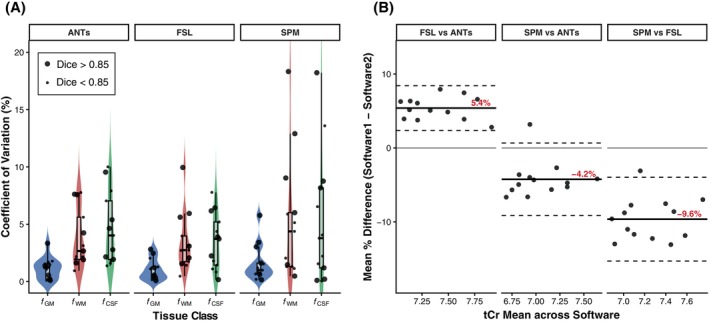
(A) Test–retest variability of tissue fractions across software tools. Violin plots show each participant's coefficient of variation (CV [%]) between sessions 1 and 2 for estimated voxel fractions of gray matter (*f*_GM_), white matter (*f*_WM_), and CSF (*f*_CSF_), obtained using ANTs, FSL, and SPM. Point size reflects the Dice overlap coefficient between sessions, with larger markers indicating greater spatial overlap of voxels. (B) Bland–Altman plots of between‐tool agreement for tissue‐corrected tCr concentration estimates. Differences in tissue‐corrected tCr concentration estimates between tissue segmentation software tools (FSL vs. ANTs, SPM vs. ANTs, and SPM vs. FSL) are plotted against their means. Solid lines indicate the mean bias, and dashed lines represent the 95% limits of agreement.

Three test–retest reliability analysis permutations were evaluated: structural‐only (segmentation varies; MRS fixed), MRS‐only (MRS varies; segmentation fixed), and combined (both vary). Table 3 summarizes descriptive reliability metrics (CV_ws_, CV_bs_, and ICC) for tissue‐corrected tCr. Across segmentation tools, structural‐only permutations showed high test–retest reliability (CV_ws_ < 2%; ICCs > 0.8). MRS‐only permutations exhibited slightly higher CV_ws_ and lower ICCs for tCr. In contrast, the combined condition yielded the highest overall variability (CV_ws_ up to ∼3%) and correspondingly lower ICCs (∼0.5), representing the reliability of the full analysis pipeline. The distributions of CVs in the combined condition across tissue classes and segmentation tools are shown in Figure 3A. Across tools, GM exhibited the lowest CVs, with SPM showing higher CVs for both WM and CSF. Dice overlap coefficients indicated generally high voxel placement reproducibility across sessions (Figure S5), with most participants showing Dice values in the ∼0.7–0.9 range.

**TABLE 3:** Average coefficients of variation (mean CV ± SD [%]) and intraclass correlation coefficients (ICC [95% CI]) for tCr concentration estimates under three test–retest analysis permutations: structural‐only (segmentation varies, MRS fixed), MRS‐only (MRS varies, segmentation fixed), and combined (both vary).

		CV_ws_			CV_bs_			ICC		
Permutation	Source of variability	ANTs	FSL	SPM	ANTs	FSL	SPM	ANTs	FSL	SPM
Structural‐only	Segmentation	1.28 ± 0.89	1.19 ± 0.77	1.15 ± 1.1	4.17 ± 0.30	4.19 ± 0.31	4.63 ± 0.32	0.87 (0.63–0.96)	0.86 (0.62–0.95)	0.86 (0.63–0.95)
MRS‐only	Fitting and signal properties	1.86 ± 1.76	1.86 ± 1.76	1.86 ± 1.76	4.14 ± 0.29	3.72 ± 0.28	4.37 ± 0.30	0.68 (0.24–0.89)	0.68 (0.23–0.89)	0.74 (0.34–0.91)
Combined	Full pipeline	2.63 ± 2.00	2.61 ± 2.00	2.74 ± 2.30	4.04 ± 0.29	3.85 ± 0.29	4.18 ± 0.28	0.49 (0.06–0.81)	0.46 (0.11–0.79)	0.46 (0.12–0.80)

*Note*: CV_ws_ denotes the within‐subject variation, whereas CV_bs_ denotes the between‐subject variation.

Pairwise ICC comparisons (each tool tested against the competing tool's ICC) yielded no additional significant differences (Table S5). Test–retest agreement for tissue‐corrected tCr estimates is illustrated in Figure S6. Bland–Altman plots showed negligible systematic bias across sessions for all segmentation tools, with most differences falling within the 95% limits of agreement. Between‐tool comparisons (Figure 3B) showed similarly small biases and tight limits of agreement, with slightly greater deviations observed for SPM relative to ANTs and FSL.

All variance partitioning analyses reported here were performed for tissue‐corrected tCr (variance components may differ for other metabolites). For the VPCs, session effects accounted for negligible variance (< 1%) and were therefore removed from the linear mixed‐effects models. Based on the recalculated VPCs, segmentation tool‐ and participant‐related factors accounted for the majority of variance across permutations. Residual variance increased with greater pipeline complexity. VPCs are provided in Table 4.

**TABLE 4:** Summary of variance partition analyses for tCr concentration estimates for the three test–retest analysis permutations.

	Structural‐only	MRS‐only	Combined
Segmentation tool	56.8%	50.0%	51.3%
Participant	34.7%	36.2%	28.5%
Residual	8.5%	13.8%	20.2%

### 3.5 Experiment 2: Segmentation Agreement With Manual Labels and Perturbation Robustness

**FIGURE 4:**
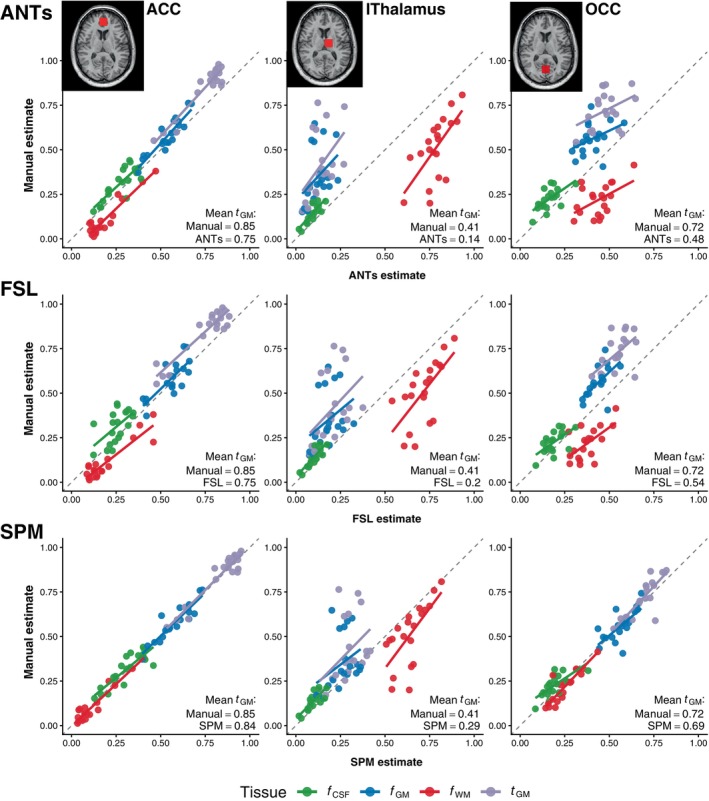
Agreement between tissue‐fraction estimates from manual and software segmentation across tools and brain ROIs using the benchmark MRBrainS dataset in Experiment 2. Each panel shows the relation between manual tissue fraction estimates and the corresponding estimates from ANTs, FSL, and SPM. Within each panel, data are displayed for each ROI positioned in the anterior cingulate cortex (ACC; left column), left thalamus (middle column), and occipital cortex (OCC; right column).

Table 5 summarizes the ICCs for each segmentation tool relative to the manual reference, ranging from 0.09 to 0.96 across ROIs, tissue classes, and tools, with the lowest values observed for GM. The robustness test comparing original and perturbed manual masks showed no significant ICC differences after Bonferroni correction (Table S6), indicating that the perturbation had a negligible effect. Manual‐versus‐software segmentation agreement showed strong region dependence (Figure 4). In the ACC, all three segmentation tools produced tissue fractions that closely tracked the manual labels, with data points tightly clustered around the identity line across tissue classes. In contrast, the left thalamus showed greater disagreement. The OCC showed generally good correspondence, but there were noticeable offsets for select tissue classes, depending on the tool.

**TABLE 5:** Intraclass correlation coefficients (ICC) and 95% confidence intervals (CIs) comparing tissue fraction estimates from manual and software segmentation across ROIs.

ROI	Tissue class	SPM	FSL	ANTs
ACC	GM	0.92 (0.82–0.97)	0.76 (0.49–0.90)	0.83 (0.28–0.95)
	WM	0.96 (0.89–0.98)	0.72 (−0.07 to 0.92)	0.73 (−0.06 to 0.93)
	CSF	0.87 (0.70–0.94)	0.42 (−0.03 to 0.72)	0.76 (0.33–0.91)
lThalamus	GM	0.22 (−0.14 to 0.56)	0.13 (−0.11 to 0.45)	0.09 (−0.09 to 0.35)
	WM	0.38 (−0.08 to 0.71)	0.24 (−0.10 to 0.61)	0.17 (−0.08 to 0.51)
	CSF	0.58 (−0.09 to 0.85)	0.57 (−0.09 to 0.85)	0.47 (−0.10 to 0.81)
OCC	GM	0.70 (0.39–0.87)	0.23 (−0.09 to 0.60)	0.13 (−0.08 to 0.44)
	WM	0.82 (0.40–0.94)	0.21 (−0.08 to 0.58)	0.12 (−0.06 to 0.42)
	CSF	0.60 (0.12–0.84)	0.51 (0.06–0.78)	0.41 (−0.10 to 0.75)

### 3.6 Effect of Defacing Structural Images

Table S7 lists the mean GM/WM/CSF fractions from each defaced workflow relative to the original images. ANTs yielded identical fractions (all differences = 0.0000), FSL showed small shifts (FSL: *f*_GM_ = −0.0034, *f*_WM_ = +0.0015, *f*_CSF_ = −0.0049), and SPM showed modest changes across tissues (SPM: *f*_GM_ = −0.0073, *f*_WM_ = +0.0129, *f*_CSF_ = −0.0056). Paired *t*‐test results with Bonferroni‐adjusted *p*‐values across the three software packages are provided in Table S8. Table S9 reports the resulting differences in tissue‐corrected tCr concentrations: ANTs produced identical values, while FSL and SPM yielded significant differences (*t*(12) = 2.63; adjusted *p* = 0.04; *t*(12) = 3.75; adjusted *p* = 0.01, respectively).

## 4 Discussion

This study evaluated how brain tissue segmentation software tools influence tissue‐corrected metabolite concentration estimates quantified by in vivo MRS. Tissue‐fraction differences were evaluated for their propagation into metabolite concentrations in an in vivo test–retest dataset, and automated tools were benchmarked against manual segmentation across multiple brain regions. In Experiment 1, tissue fraction estimates were stable within each routine across sessions but differed systematically between tools, leading to significant changes in concentration estimates. In Experiment 2, agreement with manually derived tissue fractions varied widely across regions and tools. These findings have important implications for cross‐MRS study comparability, as they can alter reported normative ranges and thresholds for pathology, complicating meta‐analyses and clinical translational potential.

### 4.1 Segmentation Tool Dependence and Error Propagation Into Absolute Metabolite Concentration Estimates

Segmentation methodology influences estimated absolute metabolite concentrations, as many contemporary MRS processing pipelines leverage established neuroimaging tools such as ANTs, FSL, and SPM [32, 48, 49]. The impact depends strongly on study design. Within‐participant comparisons (e.g., task versus rest, pre‐ versus post‐intervention) are likely to be the least affected by segmentation variability, as tissue misclassification errors may cancel out when anatomical structures and voxel placement are consistent across conditions. In contrast, within‐study, between‐participant comparisons, such as group differences, may be moderately impacted, especially in cohorts with greater anatomical variability due to aging or pathology. In these cases, systematic biases in tissue proportion estimates as a function of anatomy could distort group‐level inferences. Longitudinal studies, particularly those spanning developmental or degenerative changes, may also be susceptible, as shifts in anatomy over time could interact with segmentation biases in complex ways. The greatest vulnerability arises in cross‐study comparisons attempting to establish estimated absolute concentrations as the primary outcome, for example, quantitative biomarker thresholds. Here, the choice of segmentation tool can yield differences of several mM in metabolite estimates, even when acquisition, processing, modeling, and relaxation correction parameters are otherwise identical, posing significant challenges for data harmonization, meta‐analyses, and clinical translation. Segmentation‐tool dependence is not unique to MRS: in modalities that use MR‐derived tissue maps for partial‐volume correction (e.g., ASL and PET) [50, 51], segmentation and registration choices can propagate to corrected quantitative outcomes and hinder cross‐pipeline comparability. In practice, this can be managed through pipeline standardization and quality control within studies, and, when cross‐study comparability is required, reprocessing through common workflows.

Tissue segmentation affects the estimation of absolute concentration, as tissue fractions directly inform partial‐volume and relaxation corrections of the water signal based on literature values. In particular, the estimated CSF fraction strongly influences the derived concentrations due to partial‐volume correction and substantially longer CSF relaxation times and can introduce a significant bias if inaccurately determined. Metabolite concentration estimates rely on assumed values for tissue‐specific relaxation times and water content, often derived from literature averages that may not reflect individual physiology. While this is not the focus of the present study, it represents a fundamental constraint on the precision of absolute quantification estimates, further compounding the variability introduced by segmentation. Finally, we note that there are also tissue‐dependent differences in metabolite relaxation times and in intrinsic metabolite concentrations (i.e., metabolites are inherently more concentrated in some tissues than others). Such corrections have been considered before [36, 52, 53], but were not applied in this study to avoid over‐complicating the scope of our investigation.

### 4.2 Reliability and Sources of Variance

Across tools, test–retest reliability was comparable, with similar CVs and overlapping ICC confidence intervals for tCr. The between‐subject CV of ∼3% for tCr lies within the commonly reported test–retest reproducibility for major singlet metabolites (< 5%) [54, 55]. Variance partitioning further indicated that, under a fixed MRS acquisition condition, segmentation tool accounted for a substantial proportion of the observed variance (57%). In this scenario, segmentation‐induced differences behave as a shared scaling effect across metabolites; accordingly, tissue‐corrected tCr is reported as a representative example. Allowing the MRS component to vary reduced reliability (ICC≈0.4) and increased (CV≈2.6%), indicating that fitting and quantification steps represent a substantial source of variance in the full pipeline (along with other biological processes). Consistent with this interpretation, prior work has shown that linear‐combination modeling choices can yield systematically different metabolite estimates from the same spectra [56], with agreement varying by metabolite (e.g., more consistent for tNAA and tCho than for Glx and mI in some comparisons). Notably, reliability reflects consistency rather than accuracy [15], yet without ground‐truth validation (e.g., histologically informed or post‐mortem comparisons), reliability remains an informative metric. Resources such as the next‐generation NextBrain atlas (a probabilistic histological human brain atlas released with a Bayesian MRI segmentation tool) may make histology‐linked accuracy benchmarking increasingly tractable [57]. Together, these findings emphasize that reproducibility and cross‐study comparability require transparent reporting of segmentation tools as well as preprocessing, fitting, and quantification settings.

### 4.3 Manual Tissue Segmentation and Region Dependence

Evaluation of manual tissue segmentation using the MRBrainS dataset showed that agreement between automated tissue fractions and manual tissue labeling is strongly region dependent, suggesting that segmentation performance across ROIs varies. SPM showed the highest agreement across regions, in line with prior reports [26], and uniformly low concordance across tools was observed in the thalamus. Although metabolite quantification was not performed in Experiment 2, the regional and tool‐specific structure of these segmentation discrepancies suggests that partial‐volume correction of absolute metabolite concentration estimates may be affected by both segmentation approach and anatomy. This pattern is consistent with known challenges in subcortical segmentation [58], including reduced tissue contrast, complex boundaries, susceptibility to partial‐volume effects, and sensitivity to registration and atlas priors. Importantly, the intent of this experiment was not to identify a single best tool, but to demonstrate that segmentation error is systematic and region‐specific within volumes commonly used in MRS studies.

### 4.4 Exploratory Analysis on Defacing Effects of Structural Images

Overall, the defacing‐related differences were numerically small (e.g., tissue‐fraction differences on the order of ∼0.3%–1.3% and tCr differences of ∼0.05 mM), with ANTs showing no measurable impact and FSL and SPM producing statistically significant but likely practically negligible differences relative to typical MRS measurement uncertainty. If facial‐identifiability risk is a key concern, an alternative is to use a “refacing” approach (e.g., mri_reface) [59] that replaces the face rather than removing it.

### 4.5 Limitations and Future Directions

The absence of a definitive ground‐truth tissue segmentation complicates the assessment of absolute accuracy across tools, as even manual segmentations can be subjective and introduce bias. Although the structural‐only test–retest analysis permutation used MRI from separate sessions, voxel placement variability was small (Dice≈0.8), and the tissue fractions from each MRI were used directly in the quantification. Nevertheless, future work could include overlap metrics as covariates. Moreover, the influence of skull‐stripping algorithms warrants systematic evaluation, as inaccurate brain masks can propagate to tissue segmentation errors, particularly when the MRS voxel is positioned near the skull, where boundary definitions are more challenging [26]. Future work should also develop analytical frameworks for evaluating segmentation tools. Mathematical modeling approaches, such as uncertainty and sensitivity analyses, could quantify how segmentation variability propagates into MRS quantification outcomes under controlled conditions [60].

## 5 Conclusions

The impact of tissue segmentation by three widely used software tools on metabolite concentration estimates was assessed using a test–retest MR dataset. We observed significant differences in tCr measurements that were independent of session effects. Reliability was comparable across tools, suggesting that in the absence of an in vivo ground truth, any tested approach is acceptable if applied consistently and reported transparently. These results underscore that segmentation choice influences quantitative MRS results, especially across studies, and that standardization would improve reproducibility, harmonization, and cross‐study comparability. More broadly, they motivate open data‐sharing practices, such as using public repositories for MRS data and code, to enable reanalysis under a common segmentation pipeline or controlled head‐to‐head comparisons under identical conditions.

## Funding

This work was supported by NIH grant K99EB028828. CCF is supported by NIH grant DP2NS127704. GO and the Osprey software development are supported by NIH grants R01EB035529 and R21EB033516. GO is a paid consultant for Neurona Therapeutics Inc. (unrelated to this work). HJZ is supported by NIH grant K99AG080084.

## Supporting information


**Figure S1.** Representative structural image quality across datasets from Experiments 1 and 2. Example axial *T*_1_‐weighted images slices from each dataset are shown to illustrate the typical gray matter/white matter (GM/WM) contrast and overall image quality used for tissue segmentation analyses.
**Figure S2.** Density plots of metabolite concentration estimates by dataset (segmentation tool), faceted by metabolite. Distributions are shown for each metabolite (e.g., tNAA, tCr, tCho, Glu, Glx, mI), with density curves overlaid for each segmentation tool (ANTs, FSL, and SPM). These plots illustrate the variability and distributional characteristics across tools. Normality was statistically assessed using the Shapiro–Wilk test within each dataset, session, and metabolite, with Bonferroni‐adjusted *p*‐values reported in Table S1.
**Figure S3.** MRIQC quality metrics by session. Each violin plot shows the distribution of quality metric values across sessions, with individual data points overlaid. No significant differences were observed between sessions (CNR: *t* = 0.05, *p* = 0.96; total SNR: *t* = 0.18, *p* = 0.86; Bonferroni‐adjusted).
**Figure S4.** Metabolite concentrations (mM) across the three segmentation tools (ANTs, FSL, and SPM). Inferential statistics were performed using repeated‐measures ANOVAs with Tukey post hoc‐corrected pairwise comparisons. tNAA, total *N*‐acetylaspartate; tCho, total choline; mI, *myo*‐inositol, Glu, glutamate; Glx, glutamate + glutamine. ** *p* < 0.01, ***p < 0.001.
**Figure S5.** Voxel placement reproducibility across test–retest sessions. For each participant, spatial overlap between voxel masks from sessions 1 and 2 is quantified by the Dice overlap coefficient (reported in the panel titles; Dice = 1 indicates perfect overlap). Heatmaps display the slice‐wise difference between voxel masks (session 1−session 2) on the subject‐specific mid‐voxel slice, with nonzero values (blue and yellow) highlighting voxels present in the mask from only one session.
**Figure S6.** Bland–Altman plots of test–retest agreement for tissue‐corrected tCr levels across segmentation tools. Differences between sessions 1 and 2 tCr metabolite concentration estimates are plotted against their mean for ANTs, FSL, and SPM.
**Table S1.** Assessment of normality of the distributions of metabolite concentration estimates. Shapiro–Wilk tests were performed across all datasets (ANTS, FSL, SPM), sessions (sessions 1 and 2), and metabolites (tNAA, *N*‐acetylaspartate; tCr, total creatine; tCho, total choline; mI, *myo*‐inositol; Glu, glutamate; Glx, glutamate + glutamine).
**Table S2.** MRSinMRS checklist^1^.
**Table S3.** Longitudinal (*T*_1_) and transverse (*T*_2_) relaxation constants used in quantification calculations^2–4^. Values are averaged over gray and white matter and metabolite moieties.
**Table S4.** Tukey‐adjusted pairwise comparisons of metabolite concentrations (mM) between segmentation tools. Significant main effects of segmentation tool were observed for all metabolites: tNAA, *F* (2, 60) = 46.07, *p* < 0.0001; mI, *F* (2, 60) = 68.10, *p* < 0.0001; Glx, *F* (2, 60) = 21.79, *p* < 0.0001; Glu, *F* (2, 60) = 27.44, *p* < 0.0001; tCho, *F* (2, 60) = 88.63, *p* < 0.0001. There were no significant interactions between segmentation tool and session for any metabolite (all *p* > 0.92), justifying the use of pairwise comparisons averaged across sessions.
**Table S5.** Pairwise ICC comparisons. Each tool was tested against the competing tool's ICC, and no additional significant differences were observed.
**Table S6.** Differences between original and perturbed ICCs across ROIs. The robustness test showed no significant differences after Bonferroni correction.
**Table S7.** Mean GM/WM/CSF fractions for original versus defaced images. Differences are reported as original—defaced.
**Table S8.** Statistical comparisons of estimated tissue fractions. Bonferroni‐corrected paired *t*‐tests comparing tissue fractions derived from original vs. defaced structural images.
**Table S9.** Statistical comparisons of tCr levels. Bonferroni‐corrected paired *t*‐tests comparing tissue‐corrected total creatine (tCr) levels based on the original vs. defaced structural MR images.

## Data Availability

All MR data and code used in this study are publicly available on OpenNeuro at https://openneuro.org/datasets/ds006444. The basis set simulation code is available on GitHub at https://github.com/arcj‐hub/BasisSetSimulation. The code for quantification and the statistical analyses is included in the OpenNeuro repository. Note that all anatomical MR images were de‐faced using PyDeface (v2.0.2, https://github.com/poldracklab/pydeface) to allow public sharing. The MRBrainS dataset is available at https://mrbrains13.isi.uu.nl/.
