# Supplementary material for: The Impact and Reliability of Tissue Segmentation on In Vivo Magnetic Resonance Spectroscopy Metabolite Quantification
Source: Magn Reson Med. 2026 Apr 10;96(2):516–29. doi: 10.1002/mrm.70380 (PMC13269192; doi:10.1002/mrm.70380)
Supplement: Supplementary file 1 — Figure S1. Representative structural image quality across datasets from Experiments 1 and 2. Example axial T 1‐weighted images slices from each dataset are shown to illustrate the typical gray matter/white matter (GM/WM) contrast and overall image quality used for tissue segmentation analyses. Figure S2. Density plots of metabolite concentration estimates by dataset (segmentation tool), faceted by metabolite. Distributions are shown for each metabolite (e.g., tNAA, tCr, tCho, Glu, Glx, mI), with density curves overlaid for each segmentation tool (ANTs, FSL, and SPM). These plots illustrate the variability and distributional characteristics across tools. Normality was statistically assessed using the Shapiro–Wilk test within each dataset, session, and metabolite, with Bonferroni‐adjusted p‐values reported in Table S1. Figure S3. MRIQC quality metrics by session. Each violin plot shows the distribution of quality metric values across sessions, with individual data points overlaid. No significant differences were observed between sessions (CNR: t = 0.05, p = 0.96; total SNR: t = 0.18, p = 0.86; Bonferroni‐adjusted). Figure S4. Metabolite concentrations (mM) across the three segmentation tools (ANTs, FSL, and SPM). Inferential statistics were performed using repeated‐measures ANOVAs with Tukey post hoc‐corrected pairwise comparisons. tNAA, total N‐acetylaspartate; tCho, total choline; mI, myo‐inositol, Glu, glutamate; Glx, glutamate + glutamine. ** p < 0.01, ***p < 0.001. Figure S5. Voxel placement reproducibility across test–retest sessions. For each participant, spatial overlap between voxel masks from sessions 1 and 2 is quantified by the Dice overlap coefficient (reported in the panel titles; Dice = 1 indicates perfect overlap). Heatmaps display the slice‐wise difference between voxel masks (session 1−session 2) on the subject‐specific mid‐voxel slice, with nonzero values (blue and yellow) highlighting voxels present in the mask from only one session. Figure S6. Bla [file MRM-96-516-s001.docx]

**The Impact and Reliability of Tissue Segmentation on In Vivo Magnetic Resonance Spectroscopy Metabolite Quantification**

Jessica Archibald^[[1]](#footnote-1)^*, Kay Chioma Igwe^2^*, Antonia Kaiser^3^, Karl Landheer^4^, Jaimie Lee^5^, John L.K. Kramer^5^, Aaron T. Gudmundson^6^*,* Helge J. Zöllner^7^, Georg Oeltzschner^7^, Candace C. Fleischer^8,9^, Niklaus Zölch^10^, Jamie Near^11^, Mark Mikkelsen^1^.

^1^ Department of Radiology, Weill Cornell Medicine, New York, NY, USA.

^2^ Department of Biomedical Engineering, Columbia University Fu Foundation School of Engineering and Applied Science, New York, NY, USA.

^3^ CIBM Center for Biomedical Imaging, École polytechnique fédérale de Lausanne, Lausanne, Switzerland.

^4^ Regeneron Genetics Center, Tarrytown, NY, USA.

^5^ Department of Anesthesiology, Pharmacology and Therapeutics, Faculty of Medicine, University of British Columbia, Vancouver, BC, Canada.

^6^ The Malone Center for Engineering in Healthcare, Johns Hopkins University, Baltimore, MD, USA.

^7^ Russell H. Morgan Department of Radiology and Radiological Science, Johns Hopkins University School of Medicine, Baltimore, MD, USA.

^8^ Department of Radiology and Imaging Sciences, Emory University School of Medicine, Atlanta, GA, USA.

^9^ Department of Biomedical Engineering, Georgia Institute of Technology and Emory University, Atlanta, GA, USA.

^10^ Institute of Forensic Medicine, Universität Zürich, Zürich, Switzerland.

^11^ Sunnybrook Research Institute and University of Toronto, Toronto, ON, Canada.

**ANTs (Advanced Normalization Tools) Atropos**

ANTs Atropos^21^ (v2.5.1) (<https://github.com/ANTsX/ANTs>) employs a Bayesian framework coupled with a non-parametric finite mixture model (FMM), which can switch between a GMM or an FMM depending on the assumed distributions of each tissue class, to optimize the voxel classification into specific tissue classes. The FMM assumes voxel-wise independence when estimating the likelihood that a voxel belongs to a specific class, based on the observed intensity across the image. To incorporate spatial coherence, Atropos integrates prior probabilities, modeled using either MRFs or user-selected template-based labeled priors. The soft expectation-maximization (EM) algorithm is then used to iteratively find the optimal voxel classifications by maximizing the posterior probability and redefining the mixing parameter, gamma-*k*, at each iteration. This balances the likelihood contribution (from the FMM or GMM) with the prior probabilities, allowing voxels to be assigned to multiple classes. For consistency in comparison, we used the same priors in ANTs as those applied in SPM12.

**FSL FMRIB’s Automated Segmentation Tool (FAST)**

FAST^22^ (v6.0.5) (<https://fsl.fmrib.ox.ac.uk/>) employs a stochastic approach by using an HMRF model with EM (HMRF-EM) and iterative conditional modes (ICM) to perform three tissue segmentations. This algorithm incorporates spatial priors, as a voxel’s membership in a specific tissue class depends on the influence of the surrounding voxels. This HMRF-EM framework employs a maximum a posteriori estimate to compute the bias field and class labels, while maximum likelihood is used to estimate the model parameters.

**Statistical Parametric Mapping (SPM)**

The SPM12^20^ (v7771) (<https://www.fil.ion.ucl.ac.uk/spm/>) unified segmentation algorithm uses a generative model that jointly performs tissue classification, intensity nonuniformity bias correction, and image registration within a probabilistic Bayesian framework. The probability that a voxel belongs to a specific class is modeled by a GMM, where the likelihood function assumes independence. Like Atropos, voxel spatial coherence is incorporated via spatial priors. The algorithm applies intensity nonuniformity bias correction to log-transformed data, also within a GMM framework. Finally, to capture voxel spatial dependence, a modified version of the International Consortium for Brain Mapping (ICBM) Tissue Probabilistic Atlas as the spatial prior is used, where a GMM approach is still used; however, each tissue is represented by its own set of Gaussian distributions, parameterizing tissue classifications through a combination of Gaussians that represent a specific tissue type. A regularization term is added to the model to penalize nonuniformity or the presence of an intensity bias field. Traditional EM is used to iteratively estimate the parameters of the probabilistic model that characterizes the underlying tissue classifications and biases in the image data. Optimizing the objective function and incorporating the regularization term makes this a parametric approach.

**Figure S1.** **Representative structural image quality across datasets from Experiments 1 and 2.** Example axial *T*_1_-weighted images slices from each dataset are shown to illustrate the typical grey matter/white matter (GM/WM) contrast and overall image quality used for tissue segmentation analyses.


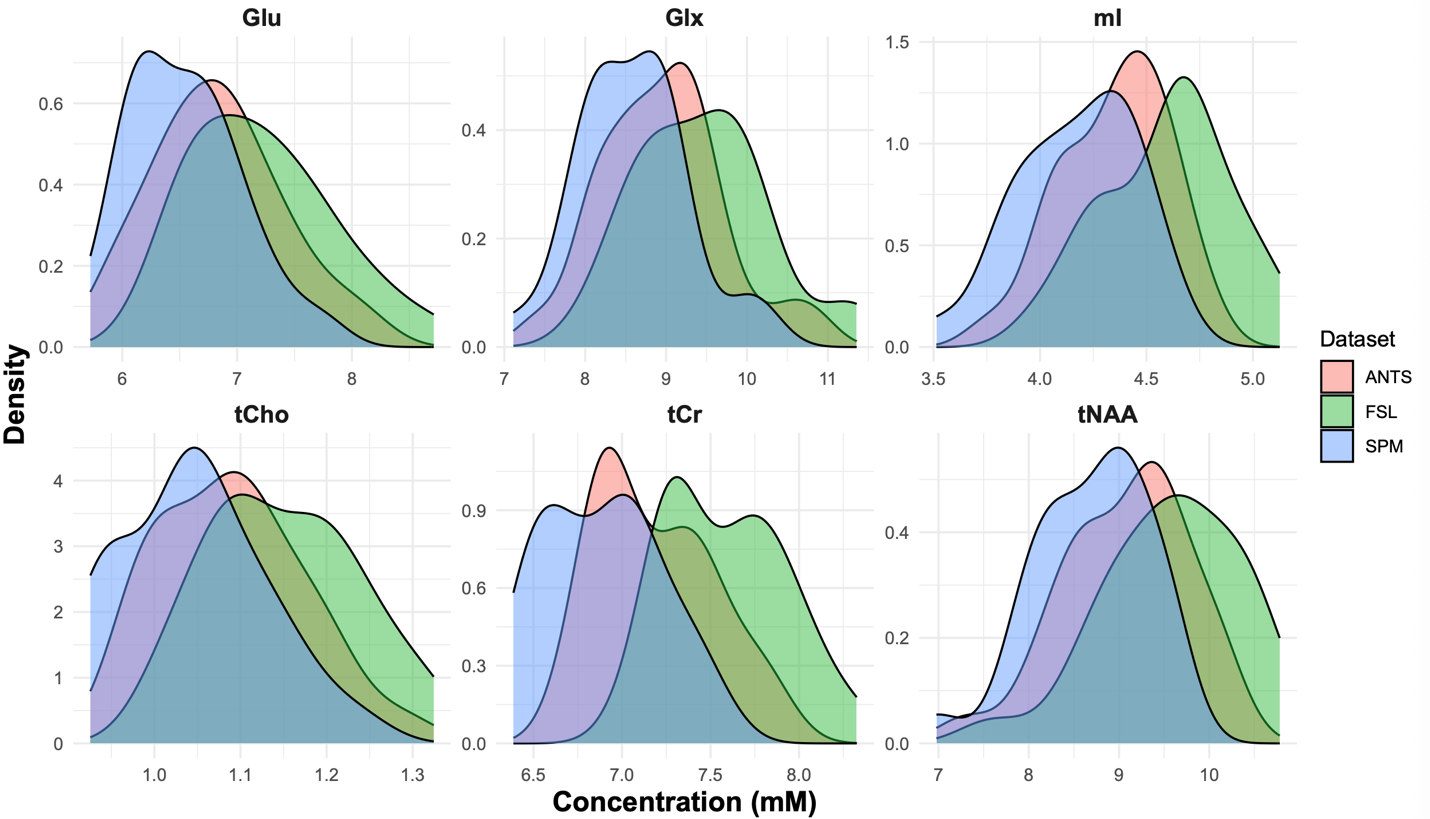


**Figure S2. Density plots of metabolite concentration estimates by dataset (segmentation tool), faceted by metabolite.** Distributions are shown for each metabolite (e.g., tNAA, tCr, tCho, Glu, Glx, mI), with density curves overlaid for each segmentation tool (ANTs, FSL, and SPM). These plots illustrate the variability and distributional characteristics across tools. Normality was statistically assessed using the Shapiro–Wilk test within each dataset, session, and metabolite, with Bonferroni-adjusted *p*-values reported in Table S1.

**
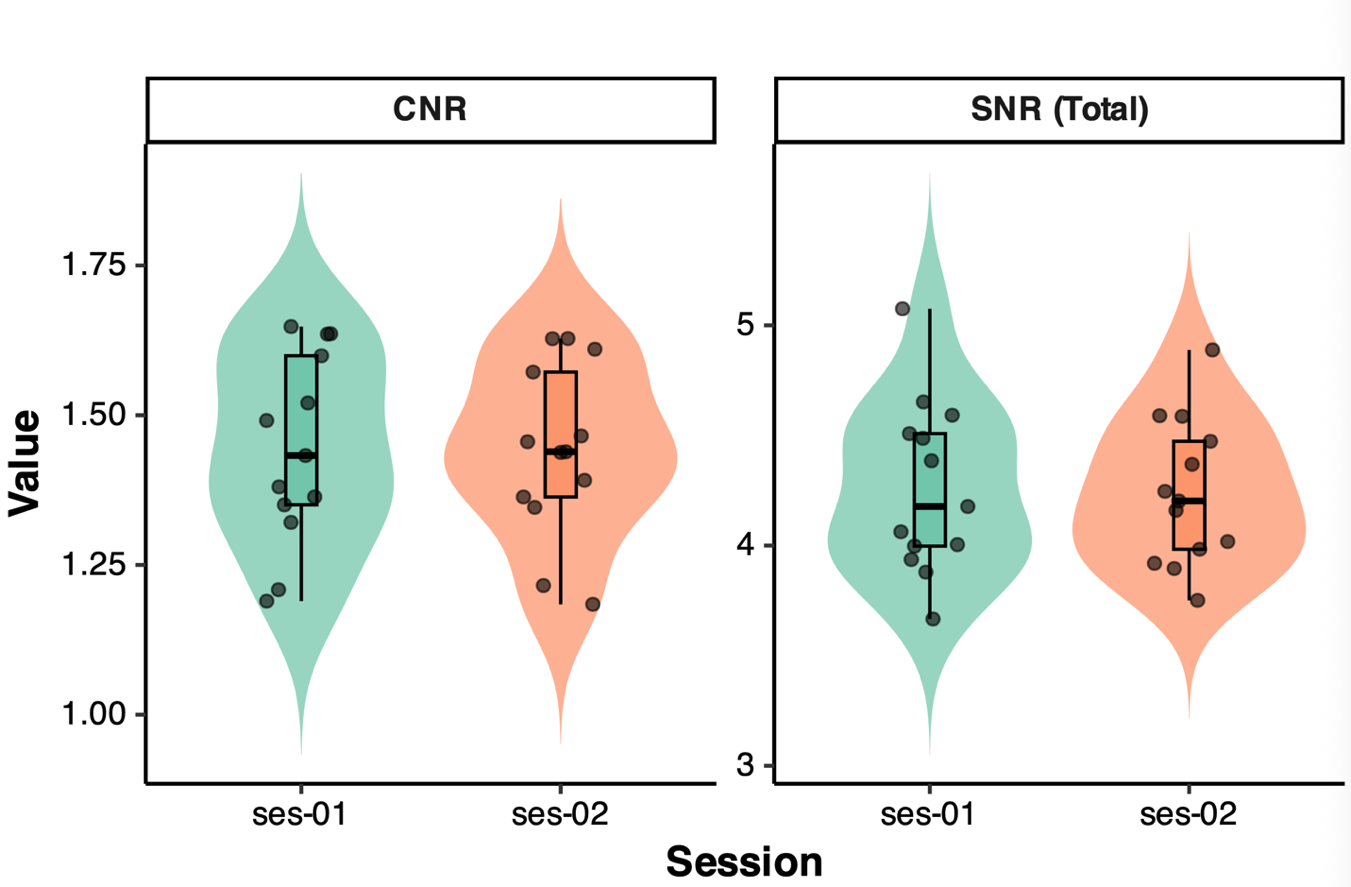
**

**Figure S3. MRIQC quality metrics by session.** Each violin plot shows the distribution of quality metric values across sessions, with individual data points overlaid. No significant differences were observed between sessions (CNR: *t* = 0.05, *p* = 0.96; total SNR: *t* = 0.18, *p* = 0.86; Bonferroni-adjusted).


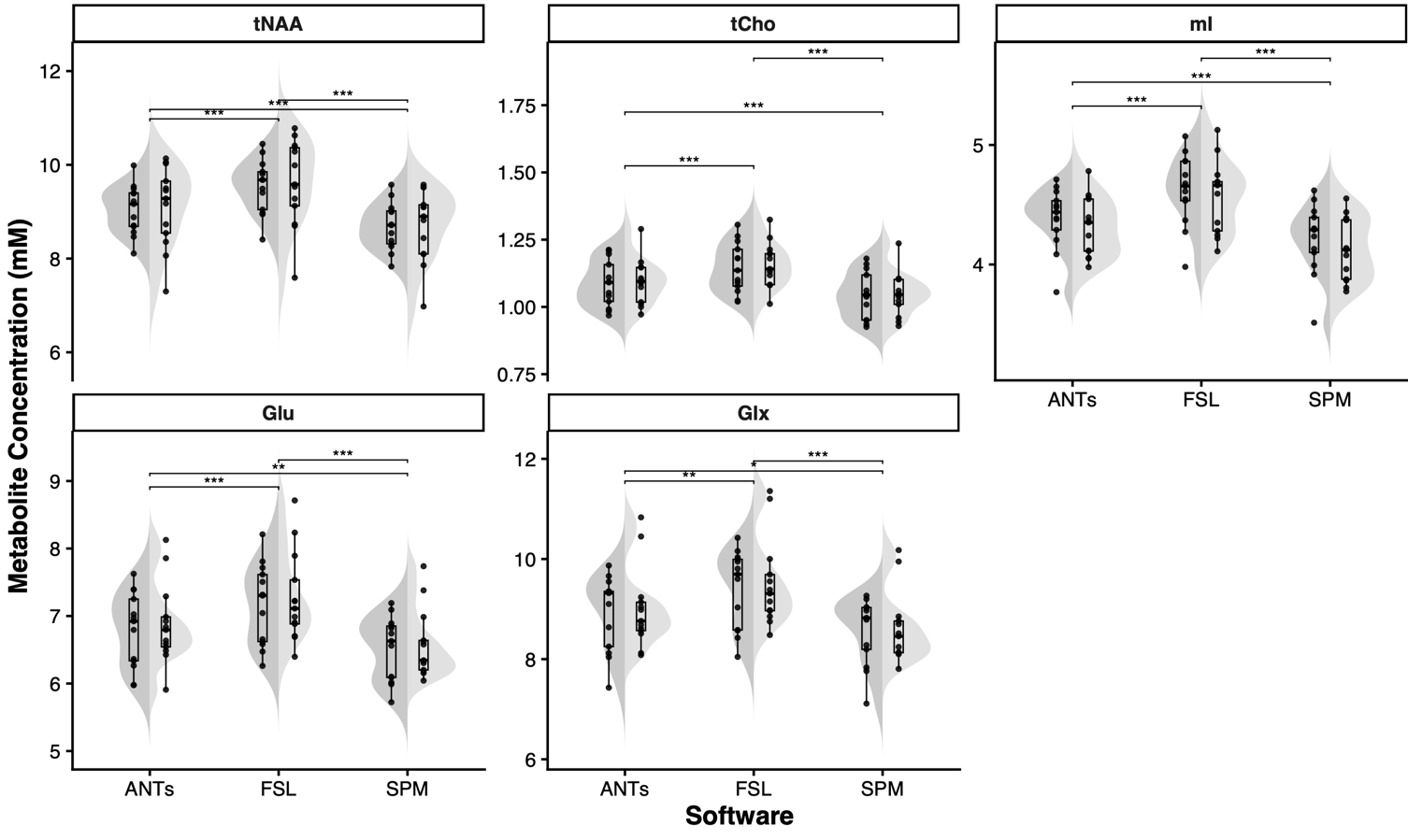


**Figure S4. Metabolite concentrations (mM) across the three segmentation tools (ANTs, FSL, and SPM).** Inferential statistics were performed using repeated-measures ANOVAs with Tukey post-hoc-corrected pairwise comparisons. tNAA, total *N*-acetylaspartate; tCho, total choline; mI, *myo*-inositol, Glu, glutamate; Glx, glutamate + glutamine. ** = *p* < 0.01, *** = *p* < 0.001.


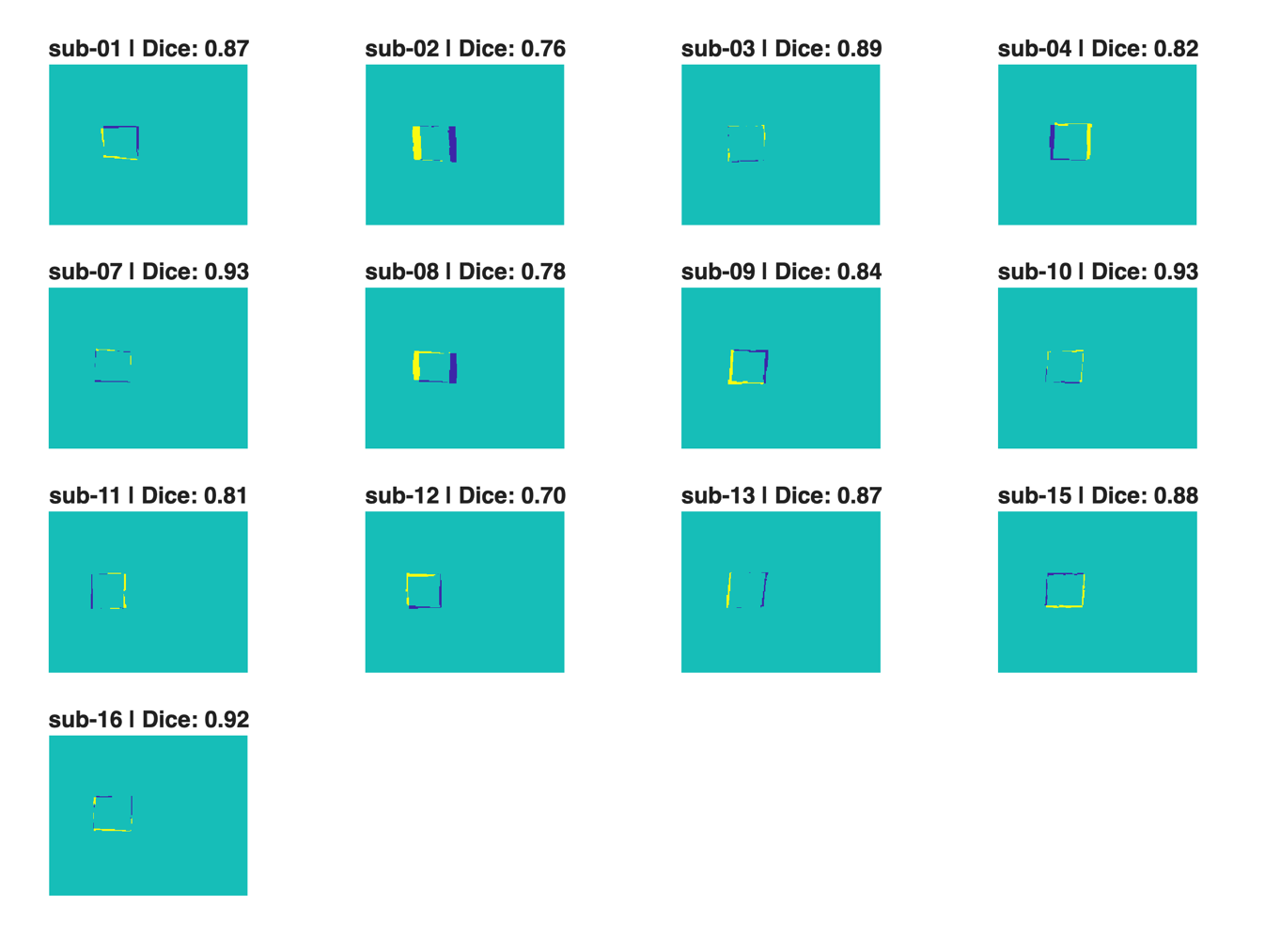


**Figure S5. Voxel placement reproducibility across test-retest sessions.** For each participant, spatial overlap between voxel masks from sessions 1 and 2 is quantified by the Dice overlap coefficient (reported in the panel titles; Dice = 1 indicates perfect overlap). Heatmaps display the slice-wise difference between voxel masks (session 1 − session 2) on the subject-specific mid-voxel slice, with nonzero values (blue and yellow) highlighting voxels present in the mask from only one session.


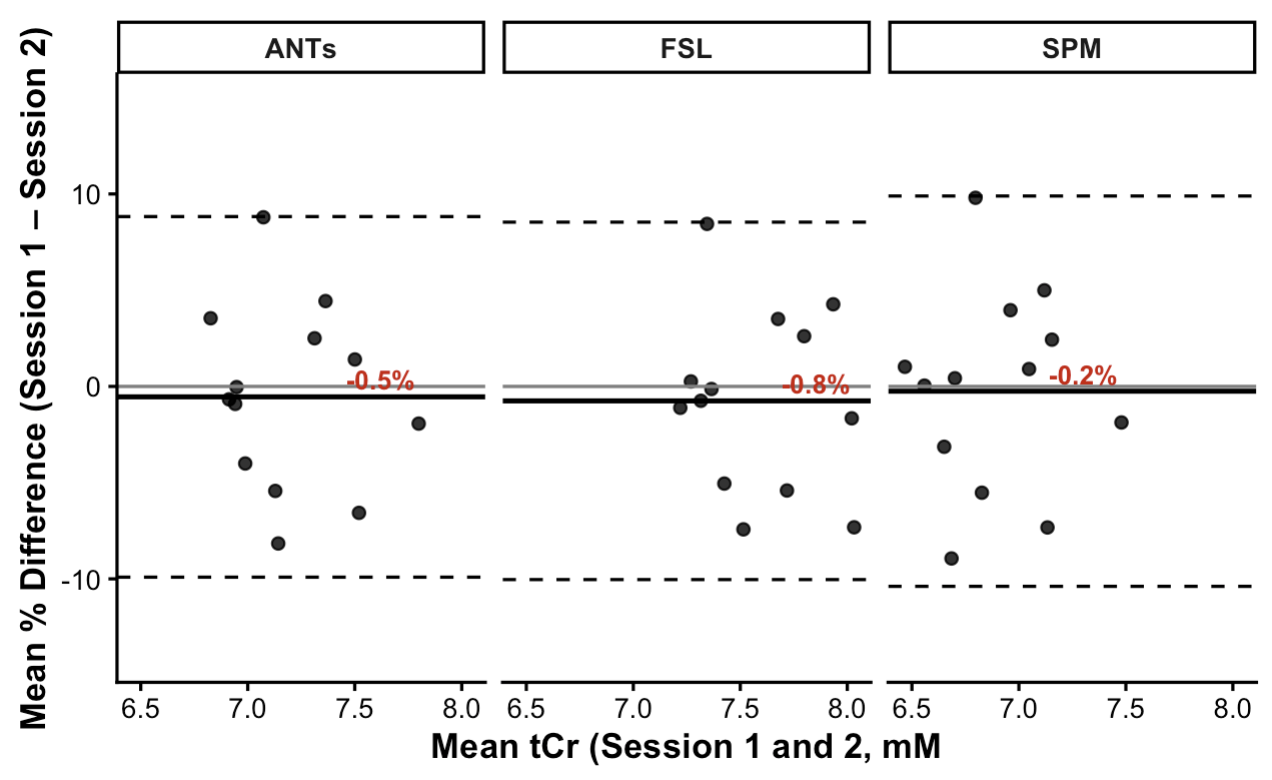


**Figure S6. Bland–Altman plots of test-retest agreement for tissue-corrected tCr levels across segmentation tools.** Differences between sessions 1 and 2 tCr metabolite concentration estimates are plotted against their mean for ANTs, FSL, and SPM.

**Table S1.** **Assessment of normality of the distributions of metabolite concentration estimates.** Shapiro–Wilk tests were performed across all datasets (ANTS, FSL, SPM), sessions (sessions 1 and 2), and metabolites (tNAA, *N*-acetylaspartate; tCr, total creatine; tCho, total choline; mI, *myo*-inositol; Glu, glutamate; Glx, glutamate + glutamine).

| **Dataset** | **Session** | **Metabolite** | **Adjusted p-value** |
| --- | --- | --- | --- |
| **ANTS** | 1 | Glu | >0.99 |
|  | 1 | Glx | > 0.99 |
|  | 1 | mI | > 0.99 |
|  | 1 | tCho | > 0.99 |
|  | 1 | tCr | 0.95 |
|  | 1 | tNAA | > 0.99 |
|  | 2 | Glu | > 0.99 |
|  | 2 | Glx | 0.94 |
|  | 2 | mI | > 0.99 |
|  | 2 | tCho | > 0.99 |
|  | 2 | tCr | > 0.99 |
|  | 2 | tNAA | > 0.99 |
| **FSL** | 1 | Glu | > 0.99 |
|  | 1 | Glx | > 0.99 |
|  | 1 | mI | > 0.99 |
|  | 1 | tCho | > 0.99 |
|  | 1 | tCr | > 0.99 |
|  | 1 | tNAA | > 0.99 |
|  | 2 | Glu | > 0.99 |
|  | 2 | Glx | > 0.99 |
|  | 2 | mI | > 0.99 |
|  | 2 | tCho | > 0.99 |
|  | 2 | tCr | > 0.99 |
|  | 2 | tNAA | > 0.99 |
| **SPM** | 1 | Glu | > 0.99 |
|  | 1 | Glx | > 0.99 |
|  | 1 | mI | > 0.99 |
|  | 1 | tCho | > 0.99 |
|  | 1 | tCr | > 0.99 |
|  | 1 | tNAA | > 0.99 |
|  | 2 | Glu | > 0.99 |
|  | 2 | Glx | 0.51 |
|  | 2 | mI | > 0.99 |
|  | 2 | tCho | > 0.99 |
|  | 2 | tCr | > 0.99 |
|  | 2 | tNAA | > 0.99 |

**Table S2.** **MRSinMRS checklist^1^**

| 1. Hardware |  |
| --- | --- |
| a. Field strength [T] | 3T |
| b. Manufacturer | GE |
| c. Model (software version if available) | Discovery MR750 (DV26.0_R01_1725.a) |
| d. RF coils: nuclei (transmit/ receive), number of channels, type, body part | ^1^H 32-channel phased-array head coil for receive; body coil for transmit |
| e. Additional hardware | n/a |
| 2. Acquisition |  |
| a. Pulse sequence | sLASER |
| b. Volume of Interest (VOI) locations | Medial parietal lobe |
| c. Nominal VOI size | 30 × 30 × 30 mm^3^ |
| d. Repetition Time (TR), Echo Time (TE) | TR = 2000 ms; TE = 35 ms |
| e. Total number of Excitations or acquisitions per spectrum | 64 |
| f. Additional sequence parameters | Spectral width: 5000 Hz  Spectral data points: 4096  GOIA‑WURST refocusing pulse; duration/bandwidth: 4.5 ms/10 kHz |
| g. Water Suppression Method | VAPOR; pulse duration/bandwidth: 30 ms/70 Hz |
| h. Shimming Method, reference peak, and thresholds for “acceptance of shim” chosen | ﻿Double-echo GRE |
| i. Triggering or motion correction method | n/a |
| 3. Data analysis methods and outputs |  |
| a. Analysis software | Osprey (v2.9.6); LCModel (embedded in Osprey) (v6.3-1N); R (v4.5.0) |
| b. Processing steps deviating from quoted reference or product | RF coil combination performed using generalized least squares (An et al., 2013, doi:﻿[10.1002/jmri.23941](https://www.doi.org/10.1002/jmri.23941))  Basis set created for TE = 35 ms sLASER using FID-A (Simpson et al., 2017, doi:10.1016/j.neuroimage.2017.02.058), simulating 20 metabolites, LW = 2 Hz, spectral width = 5000 Hz, 64×64 grid. |
| c. Output measure  Processing steps deviating from quoted reference or product | Tissue- and relaxation-corrected “absolute” concentration estimates (mM) using internal tissue water as a concentration reference |
| d. Quantification references and assumptions, fitting model assumptions | Unsuppressed water used as a reference; model assumptions were as set by Osprey by default |
| 4. Data Quality |  |
| a. Reported variables  (SNR, Linewidth (with reference peaks)) | Creatine SNR; H_2_O FWHM; Fit Quality Index |
| b. Data exclusion criteria | Visual inspection of spectra |
| c. Quality measures of postprocessing Model fitting (e.g., CRLB, goodness of fit, SD of residual) | Fit error calculated as the sum of squares of residuals normalized to the square of the standard deviation of the noise signal between –2 and 0 ppm and multiplied by the number of points of the residuals |
| d. Sample spectrum | Provided in the main text of the article Figure 1 |

### **Table S3. Longitudinal (*T*_1_) and transverse (*T*_2_) relaxation constants used in quantification calculations^2–4^.** Values are averaged over grey and white matter and metabolite moieties.

**(A) Metabolite *T*₁ and *T*₂ Relaxation Times (ms)**

| Metabolite | *T*₁ (ms) | Reference | *T*₂ (ms) | Reference |
| --- | --- | --- | --- | --- |
| tNAA | 1410 | Mlynárik et al. (2001) | 282.25 | Wyss et al. (2018) |
| tCr | 1350 | Mlynárik et al. (2001) | 146.75 | Wyss et al. (2018) |
| tCho | 1190 | Mlynárik et al. (2001) | 241.71 | Wyss et al. (2018) |
| mI | 1090 | Mlynárik et al. (2001) | 202.5 | Wyss et al. (2018) |
| Glu | 1220 | Mlynárik et al. (2001) | 129.5 | Wyss et al. (2018) |
| Glx | 1080 | Mlynárik et al. (2001) | 129.5 | Wyss et al. (2018) |

#### **B) Water Relaxation Attenuation Factors**

| Tissue Type | *T*₁ (ms) | *T*₂ (ms) | *R*_H₂O,_*_x_*^*^ | Source |
| --- | --- | --- | --- | --- |
| Gray matter | 1331 | 110 | 0.565 | Dhamala et al. (2019) |
| White matter | 832 | 79.2 | 0.584 | Dhamala et al. (2019) |
| CSF | 3817 | 503 | 0.380 | Dhamala et al. (2019) |
| ^*^ Computed assuming TE/TR = 35/2000 ms | | | | |

**Table S4.** **Tukey-adjusted pairwise comparisons of metabolite concentrations (mM) between segmentation tools.** Significant main effects of segmentation tool were observed for all metabolites: tNAA, *F*(2, 60) = 46.07, *p* < .0001; mI, *F*(2, 60) = 68.10, *p* < .0001; Glx, *F*(2, 60) = 21.79, *p* < .0001; Glu, *F*(2, 60) = 27.44, *p* < .0001; tCho, *F*(2, 60) = 88.63, *p* < .0001. There were no significant interactions between segmentation tool and session for any metabolite (all *p* > 0.92), justifying the use of pairwise comparisons averaged across sessions.

| **Metabolite** | **Contrast** | **Mean diff.** | **Std. error** | **df** | **t** | **p-value** |
| --- | --- | --- | --- | --- | --- | --- |
| **tNAA** | ANTS – FSL | –0.51 | 0.09 | 60 | –5.47 | < 0.0001 |
|  | ANTS – SPM | 0.38 | 0.09 | 60 | 4.10 | 0.0004 |
|  | FSL – SPM | 0.88 | 0.09 | 60 | 9.57 | < 0.0001 |
| **mI** | ANTS – FSL | –0.24 | 0.04 | 60 | –6.70 | < 0.0001 |
|  | ANTS – SPM | 0.18 | 0.04 | 60 | 4.92 | < 0.0001 |
|  | FSL – SPM | 0.42 | 0.04 | 60 | 11.63 | < 0.0001 |
| **Glx** | ANTS – FSL | –0.50 | 0.13 | 60 | –3.75 | 0.0012 |
|  | ANTS – SPM | 0.38 | 0.13 | 60 | 2.84 | 0.0169 |
|  | FSL – SPM | 0.87 | 0.13 | 60 | 6.58 | < 0.0001 |
| **Glu** | ANTS – FSL | –0.38 | 0.09 | 60 | –4.20 | 0.0003 |
|  | ANTS – SPM | 0.29 | 0.09 | 60 | 3.18 | 0.0064 |
|  | FSL – SPM | 0.67 | 0.09 | 60 | 7.39 | < 0.0001 |
| **tCho** | ANTS – FSL | –0.06 | 0.01 | 60 | –7.60 | < 0.0001 |
|  | ANTS – SPM | 0.05 | 0.01 | 60 | 5.67 | < 0.0001 |
|  | FSL – SPM | 0.11 | 0.01 | 60 | 13.27 | < 0.0001 |

**Table S5.** **Pairwise ICC comparisons.** Each tool was tested against the competing tool’s ICC, and no additional significant differences were observed.

**Permutation 1**

| **Pair Tested** | **ICC (H_0_)** | **ICC** | **95% CI** | **p-value** |
| --- | --- | --- | --- | --- |
| **ANTs vs. FSL** | 0.87 (FSL) | 0.87 | [0.64, 0.96] | 0.47 |
| **ANTs vs. SPM** | 0.87 (SPM) | 0.87 | [0.64, 0.96] | 0.47 |
| **FSL vs. SPM** | 0.87 (SPM) | 0.87 | [0.62, 0.96] | 0.50 |

**Permutation 2**

| **Pair Tested** | **ICC (H_0_)** | **ICC** | **95% CI** | **p-value** |
| --- | --- | --- | --- | --- |
| **ANTs vs. FSL** | 0.68 (FSL) | 0.68 | [0.24, 0.89] | 0.49 |
| **ANTs vs. SPM** | 0.74 (SPM) | 0.68 | [0.24, 0.89] | 0.65 |
| **FSL vs. SPM** | 0.74 (SPM) | 0.68 | [0.24, 0.89] | 0.65 |

**Table S6. Differences between original and perturbed ICCs across ROIs.** The robustness test showed no significant differences after Bonferroni correction.

| **ROI** | **Tissue** | **Tool** | **ICC**  **(original)** | **ICC**  **(perturbed)** | **Difference** | **95% CI** | **Adj. p-value** |
| --- | --- | --- | --- | --- | --- | --- | --- |
| **ACC** | *f*_CSF_ | SPM | 0.99 | 0.99 | 0.00 | [–0.01, 0.01] | > 0.99 |
|  | *f*_CSF_ | FSL | 0.95 | 0.94 | 0.00 | [–0.03, 0.04] | > 0.99 |
|  | *f*_CSF_ | ANTS | 0.96 | 0.95 | 0.00 | [–0.03, 0.04] | > 0.99 |
|  | *f*_GM_ | SPM | 0.99 | 0.99 | 0.00 | [–0.01, 0.01] | > 0.99 |
|  | *f*_GM_ | FSL | 0.95 | 0.94 | 0.00 | [–0.03, 0.04] | > 0.99 |
|  | *f*_GM_ | ANTS | 0.96 | 0.95 | 0.00 | [–0.03, 0.04] | > 0.99 |
|  | *f*_WM_ | SPM | 0.99 | 0.99 | 0.00 | [–0.01, 0.01] | > 0.99 |
|  | *f*_WM_ | FSL | 0.95 | 0.94 | 0.00 | [–0.03, 0.04] | > 0.99 |
|  | *f*_WM_ | ANTS | 0.96 | 0.95 | 0.00 | [–0.03, 0.04] | > 0.99 |
| **Left**  **Thalamus** | *f*_CSF_ | SPM | 0.70 | 0.65 | 0.04 | [–0.13, 0.22] | > 0.99 |
|  | *f*_CSF_ | FSL | 0.58 | 0.54 | 0.05 | [–0.17, 0.27] | > 0.99 |
|  | *f*_CSF_ | ANTS | 0.50 | 0.45 | 0.05 | [–0.20, 0.29] | > 0.99 |
|  | *f*_GM_ | SPM | 0.70 | 0.65 | 0.04 | [–0.13, 0.22] | > 0.99 |
|  | *f*_GM_ | FSL | 0.58 | 0.54 | 0.05 | [–0.17, 0.27] | > 0.99 |
|  | *f*_GM_ | ANTS | 0.50 | 0.45 | 0.05 | [–0.20, 0.29] | > 0.99 |
|  | *f*_WM_ | SPM | 0.70 | 0.65 | 0.04 | [–0.13, 0.22] | > 0.99 |
|  | *f*_WM_ | FSL | 0.58 | 0.54 | 0.05 | [–0.17, 0.27] | > 0.99 |
|  | *f*_WM_ | ANTS | 0.50 | 0.45 | 0.05 | [0.20, 0.29] | > 0.99 |
| **OCC** | *f*_CSF_ | SPM | 0.97 | 0.96 | 0.01 | [–0.01, 0.04] | > 0.99 |
|  | *f*_CSF_ | FSL | 0.70 | 0.68 | 0.02 | [–0.16, 0.20] | > 0.99 |
|  | *f*_CSF_ | ANTS | 0.49 | 0.45 | 0.04 | [–0.21, 0.28] | > 0.99 |
|  | *f*_GM_ | SPM | 0.97 | 0.96 | 0.01 | [–0.01, 0.04] | > 0.99 |
|  | *f*_GM_ | FSL | 0.70 | 0.68 | 0.02 | [–0.16, 0.20] | > 0.99 |
|  | *f*_GM_ | ANTS | 0.49 | 0.45 | 0.04 | [–0.21, 0.28] | > 0.99 |
|  | *f*_WM_ | SPM | 0.97 | 0.96 | 0.01 | [–0.01, 0.04] | > 0.99 |
|  | *f*_WM_ | FSL | 0.70 | 0.68 | 0.02 | [–0.16, 0.20] | > 0.99 |
|  | *f*_WM_ | ANTS | 0.49 | 0.45 | 0.04 | [–0.21, 0.28] | > 0.99 |

**Table S7. Mean GM/WM/CSF fractions for original versus defaced images.** Differences are reported as original – defaced.

|  | ***f*_GM_** | | | | ***f*_WM_** | | | | ***f*_CSF_** | | | |
| --- | --- | --- | --- | --- | --- | --- | --- | --- | --- | --- | --- | --- |
|  | **Original** | **Defaced** | **Difference** | **Cohen’s *d*** | **Original** | **Defaced** | **Difference** | **Cohen’s *d*** | **Original** | **Defaced** | **Difference** | **Cohen’s *d*** |
| ***ANTs*** | 0.4496 | 0.4496 | 0.0000 | NA | 0.3313 | 0.3313 | 0.0000 | NA | 0.2190 | 0.2190 | 0.0000 | NA |
| ***FSL*** | 0.4312 | 0.4279 | 0.0034 | 0.8695 | 0.3047 | 0.3032 | 0.0015 | 0.4269 | 0.2641 | 0.2690 | –0.0049 | –0.7449 |
| ***SPM*** | 0.5623 | 0.5696 | –0.0073 | 1.6537 | 0.2692 | 0.2563 | 0.0129 | 1.7394 | 0.1685 | 0.1741 | –0.0056 | –0.9416 |

**Table S8. Statistical comparisons of estimated tissue fractions.** Bonferroni-corrected paired *t*-tests comparing tissue fractions derived from original vs. defaced structural images.

| **Tool** | **Tissue Type** | **t** | **Adj. p-value** |
| --- | --- | --- | --- |
|  | *f*_GM_ | NaN* | NaN |
| ANTs | *f*_WM_ | NaN | NaN |
|  | *f*_CSF_ | NaN | NaN |
|  | *f*_GM_ | –2.61 | 0.07 |
| FSL | *f*_WM_ | –1.10 | 0.87 |
|  | *f*_CSF_ | 2.15 | 0.15 |
|  | *f*_GM_ | 4.44 | 0.00 |
| SPM | *f*_WM_ | –8.30 | 0.00 |
|  | *f*_CSF_ | 5.03 | 0.00 |

* NaN results occur when the paired values are identical (i.e., no variance).

**Table S9. Statistical comparisons of tCr levels.** Bonferroni-corrected paired *t*-tests comparing tissue-corrected total creatine (tCr) levels based on the original vs. defaced structural MR images.

| **Tool** | **Metabolite** | **t** | **Adj. p-value** |
| --- | --- | --- | --- |
| ANTs | tCr | NaN* | NaN |
| FSL | tCr | 2.63 | 0.04 |
| SPM | tCr | 3.75 | 0.01 |

* NaN results occur when the paired values are identical (i.e., no variance).

**References**

1. Lin, A. *et al.* Minimum Reporting Standards for in vivo Magnetic Resonance Spectroscopy (MRSinMRS): Experts’ consensus recommendations. *NMR Biomed* 34, (2021).

2. Mlynrik, V., Gruber, S. & Moser, E. Proton T1 and T2 relaxation times of human brain metabolites at 3 Tesla. *NMR Biomed* 14, 325–331 (2001).

3. Wyss, P. O. *et al.* In vivo estimation of transverse relaxation time constant (T2) of 17 human brain metabolites at 3T. *Magn Reson Med* 80, 452–461 (2018).

4. Dhamala, E. *et al.* Validation of in vivo MRS measures of metabolite concentrations in the human brain. *NMR Biomed* 32, (2019).

1. * Jessica Archibald and Kay Chioma Igwe contributed equally to this work [↑](#footnote-ref-1)
